# Stellettin B renders glioblastoma vulnerable to poly (ADP-ribose) polymerase inhibitors via suppressing homology-directed repair

**DOI:** 10.1038/s41392-023-01324-8

**Published:** 2023-03-22

**Authors:** Xin Peng, Yingying Wang, Shaolu Zhang, Zhennan Tao, Yuxiang Dai, Francois X. Claret, Moshe Elkabets, Hou-Wen Lin, Zhe-Sheng Chen, Dexin Kong

**Affiliations:** 1 Tianjin Key Laboratory of Technologies Enabling Development of Clinical Therapeutics and Diagnostics, School of Pharmacy, Tianjin Medical University, Tianjin, 300070 China; 2 Key Laboratory of Immune Microenvironment and Diseases (Ministry of Education), Tianjin Medical University, Tianjin, 300070 China; 3 Department of Systems Biology, The University of Texas MD Anderson Cancer Center, Houston, TX 77030 USA; 4 Department of Neurosurgery, the Affiliated Drum Tower Hospital, School of Medicine, Nanjing University, Nanjing, 210008 China; 5 The Shraga Segal Department of Microbiology, Immunology and Genetics, Faculty of Health Sciences, Ben-Gurion University of the Negev, Beer-Sheva, 84105 Israel; 6 Research Center for Marine Drugs, State Key Laboratory of Oncogenes and Related Genes, Department of Pharmacy, Renji Hospital, School of Medicine, Shanghai Jiaotong University, Shanghai, 200127 China; 7 Department of Pharmaceutical Sciences, College of Pharmacy and Health Sciences, St. John’s University, Queens, NY 11439 USA

**Keywords:** Target identification, Drug development, CNS cancer, Drug development

**Dear Editor**,

Glioblastoma (GBM) is one of the most fatal brain tumors. Current first-line post-surgery regimens for GBM including radiotherapy and temozolomide (TMZ) chemotherapy show very limited efficacy.^1,2^ Novel therapeutic approaches for GBM patients are urgently needed.

Natural products are important sources for drug discovery, especially in the field of cancer treatment.^3^ We previously isolated stellettin B (STELB) (Fig. 1a) from marine sponge (*Jaspis stellifera*) and reported the remarkable and specific anticancer activities. Recently, a series of stellettins has been totally synthesized and the core chemical structure has been indicated.^4^ However, the specific mechanism and its role in regulating tumor biology remain largely unknown.

**Fig. 1:**
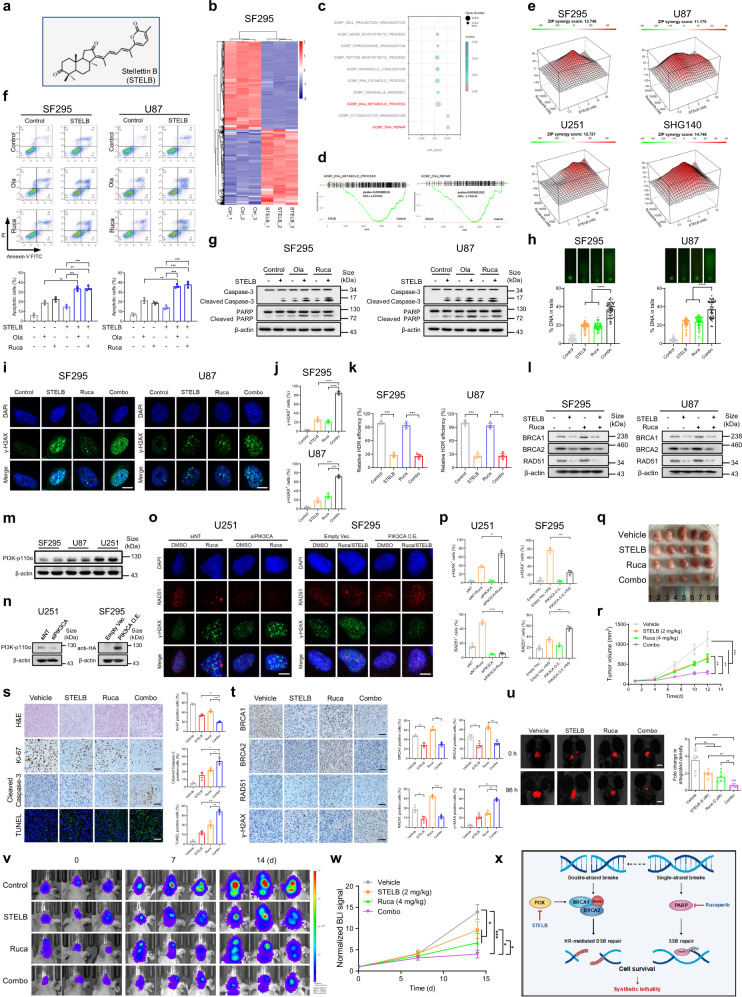
STELB sensitizes GBM to PARPis by impairing PI3K-mediated HDR. **a** Chemical Structure of STELB. **b** Hierarchical clustering shows proteins with significant difference in expression between the control group and the STELB-treated group. Values are presented as log_2_(fold change). **c** Enrichment analysis for GO biological processes in the control group vs STELB-treated group. **d** GSEA of DNA metabolic process and DNA repair in the control group vs STELB-treated group. P values and normalized enrichment score (NES) are shown. **e** ZIP model synergy test was performed in established cell lines of GBM (SF295, U87, and U251), and primary patient-derived cell line of GBM (SHG140). GBM cells were treated with increasing concentration of Olaparib/Rucaparib and STELB. After 4 days, the inhibition rate of tumor cell growth was calculated. The results are presented as ZIP synergy score with the dose-response matrix. **f** Apoptosis assays of SF295 and U87 cells incubated with STELB in combination with Olaparib or Rucaparib for 48 h. The total apoptotic cell population was calculated (Annexin V^+^/PI^+/−^ late/early apoptotic cells). **g** Western blot of PARP and Caspase-3 in SF295 and U87 cells incubated with Olaparib/Rucaparib and/or STELB for 48 h. **h** Alkaline comet assays of SF295 and U87 cells treated with Rucaparib and/or STELB for 48 h to evaluate DNA damage, the extent of which was indicated by % DNA in tails. **i** SF295 and U87 cells were incubated with Rucaparib and/or STELB for 48 h. Then the cells were subjected to γ-H2AX foci assays. Representative images are shown. Scale bar, 20 μm. **j** The percentage of γ-H2AX foci positive cells (> 5 foci/cell) is shown. **k** DR-GFP reporter assays were performed to evaluate HDR repair efficiency in GBM cells SF295 and U87. **l** Western blot of HDR-related proteins in GBM cells incubated with Rucaparib and/or STELB for 48 h. **m** Western blot of PI3K-p110α expression in SF295, U87, and U251 cells. **n** Western blot validation of siRNA knockdown of PIK3CA in U251 cells and exogenous overexpression of HA-PIK3CA in SF295 cells. **o** γ-H2AX and RAD51 foci assays of U251 cells incubated with Olaparib or Rucaparib with or without siPIK3CA, and SF295 cells incubated with STELB and Olaparib/Rucaparib with or without HA-PIK3CA. Representative images are shown. Scale bar, 20 μm. **p** The percentage of γ-H2AX/RAD51 foci positive cells are shown. **q** Subcutaneous U87 xenograft nude mice were administrated with placebo, STELB (2 mg/kg), Rucaparib (4 mg/kg), or the combination for 12 days (*n* = 5). Excised tumors are shown. **r** Tumor volumes were measured. **s** H&E, TUNEL staining, and immunohistochemistry staining of tumors of the respective groups. Statistical quantification is shown. Scale bar, 50 μm. **t** Immunohistochemistry staining of HDR-related proteins of tumors of the respective groups. Statistical quantification is shown. Scale bar, 50 μm. **u** Representative images of U87-RFP orthotopic xenograft zebrafish with various treatments, and the fluorescence intensity of the different groups are shown. Scale bar, 100 μm. **v** Representative bioluminescent images of U87 orthotopic xenograft mice are shown. **w** The relative radiance at the indicated time points was detected with a imaging system. **x** Schematic diagram of the rationale of the combination of PARPis with STELB for GBM therapy. Quantitative data are represented as mean ± SEM from three independent experiments; ns, not significant, *P < 0.05, **P < 0.01, ***P < 0.001, ****P < 0.0001

In this study, we performed unbiased proteomic analysis using label-free relative quantitative mass spectrometry to systematically monitor STELB’s effect on protein abundance profiles of GBM cell line SF295. We found 795 proteins were upregulated and 1,038 proteins were downregulated significantly (FDR < 0.05, Fig. 1b). GO analysis revealed that the biological processes (BP) of DNA metabolic process (GO: 0006259) and DNA repair (GO: 0006281) were the top enriched terms (Fig. 1c). We then conducted a gene set enrichment analysis (GSEA) and found that the involved proteins were remarkably downregulated by STELB (Fig. 1d). The abundance of main DNA repair-related proteins detected in mass spectrometry was also investigated and a concentration-dependent decrease was indicated (Supplementary Fig. 1a, b).

Poly (ADP-ribose) polymerase inhibitors (PARPis) were reported to show efficacy on ovarian and breast cancers with BRCA deficiency, but barely exert anti-GBM effects since rare BRCA mutations were found in GBM. Therefore, driven by proteomic analysis data, we speculated that STELB might sensitize GBM to PARPis by manipulating DNA repair processes.

In order to test our hypothesis, a multi-dimensional two-drug synergy assay was performed using STELB in combination with Rucaparib/Olaparib in both established- and primary patient-derived primary cell lines of GBM. Analysis of the drug interactions using the ZIP synergy model indicated a strong synergistic effect between STELB and PARPis with an average score >10 detected across 5 GBM cell lines. 3D surface plots showed regions of synergy with average ZIP synergy score, with dose-response matrix (Fig. 1e and Supplementary Fig. 2a–d). In addition, the combination of STELB and Rucaparib/Olaparib remarkably suppressed colony-forming abilities (Supplementary Fig. 3a–d) and enhanced apoptosis compared to each single treatment (Fig. 1f and Supplementary Fig. 4), as reflected by the abundance of the cleaved PARP and Caspase-3 (Fig. 1g). Using alkaline comet assays, we found that STELB and Rucaparib led to more severe DNA damage than each drug alone (Fig. 1h and Supplementary Fig. 5a). Since DNA double-strand breaks (DSBs) are the major cause of cell death induced by PARPis, we next measured γ-H2AX foci in GBM cells and confirmed a synergistic effect of STELB and Rucaparib on the accumulation of the γ-H2AX foci (Fig. 1i, j and Supplementary Fig. 5b, c). These results indicate that STELB sensitizes GBM to PARPis by augmenting DSBs.

Cells mainly rely on homology-directed repair (HDR) and Non-Homologous End Joining (NHEJ) to repair DSBs. HDR-compromised tumors are generally sensitive to PARPi. We assessed the functional effect of STELB and Rucaparib treatment on HDR and NHEJ repair efficiency. Using DR-GFP and EJ5-GFP reporter assay, we found treatment with STELB markedly reduced HDR capacity, either alone or in combination with Rucaparib in GBM cells (Fig. 1k and Supplementary Fig. 6a). This effect appears to be exclusive to the HDR as the NHEJ efficiency remained unchanged after STELB treatment (Supplementary Fig. 6b). We then observed STELB directly suppressed the protein expression of HDR factor BRCA1, BRCA2, and RAD51 (Fig. 1l and Supplementary Fig. 6c). Similar effect at the mRNA level was also found (Supplementary Fig. 6d). In addition, some key factors (Ku70 and Ku80) of NHEJ repair did not change after STELB treatment (Supplementary Fig. 6e).

To further explore how STELB affects HDR, we interrogated the PI3K axis, the key upstream regulator of the HDR process.^5^ We first found that STELB alone or in combination with Rucaparib significantly reduced the expression of PI3K and the phosphorylation of its downstream factor Akt and mTOR (Supplementary Fig. 7a). We next assessed the PI3K protein abundance in three cell lines of GBM (Fig. 1m) and examined the impact of siRNA knockdown of PIK3CA in high PI3K expressing U251 cells. We found siPIK3CA could mimic the effects of STELB on U251 cells, as reflected by the sensitization of PARPis (Supplementary Fig. 7b, c), and the enhancement of DSB level (γ-H2AX foci) with blunted HDR capacity (RAD51 foci) (Fig. 1o, p; left side). In contrast, exogenous expression of PIK3CA mitigated the repression of AKT phosphorylation and reduced the sensitivity of low PI3K expressing SF295 cells to the combined treatment of STELB and PARPis (Supplementary Fig. 7d-f), and largely reversed the effect on γ-H2AX and RAD51 foci (Fig. 1o, p; right side). These results reveal that STELB impairs HDR in a PI3K-dependent manner in GBM cells.

We then investigated the antitumor effects of Rucaparib, STELB, and the combination in vivo. With the subcutaneous U87 xenograft tumor model, we confirmed the administration of STELB alone could dose-dependently inhibit tumor growth (Supplementary Fig. 8a, b). When combined with Rucaparib, the tumor growth was delayed more strikingly than either treatment alone, without significant loss of body weight (Fig. 1q, r and Supplementary Fig. 8c, d). Using immunohistochemistry analysis, we found the combination more significantly inhibited the abundance of the Ki-67 and augmented apoptosis, as reflected by the increase of TUNEL-positive cells and cleavage of Caspase-3, in comparison to either Rucaparib or STELB alone (Fig. 1s). In addition, we found similar changes in the level of HDR-related proteins in the in vivo tumor tissues (Fig. 1t). To explore the potential of STELB to penetrate the blood-brain barrier, we utilized a U87 orthotopic xenograft zebrafish model and found that either STELB or Rucaparib administered as single agent inhibited tumor growth, and the combination inhibited much more potently (Fig. 1u). In addition, we constructed a U87 orthotopic xenograft mouse model and confirmed that the two drugs alone or in combination significantly reduced the growth of tumor, with higher potency for the combination (Fig. 1v, w), while no significant loss of body weight was indicated (Supplementary Fig. 8e), suggesting favorable anti-GBM efficacy and safety.

In conclusion, our data reveal that STELB suppresses PI3K signaling to reduce the expression of key factors of the HDR, such as BRCA1/2 and RAD51, therefore accelerating DSB accumulation caused by PARPis and leading to synthetic lethality (Fig. 1x). Since the PI3K signaling is frequently activated in GBM patients, the combination of STELB with PARPi for GBM treatment would be inherently advantageous. However, since PI3K is regulated by multiple pathways, the direct target of STELB remains unknown. Further efforts, including identification of the target with which STELB interacts, need to be made before clinical trial evaluation of STELB or its analogs for the therapy of GBM patients.

## Supplementary information

Supplementary Materials

Supplementary Table S1

## Data Availability

The mass spectrometry proteomics data have been deposited to the ProteomeXchange Consortium (http://proteomecentral.proteomexchange.org) via the iProX partner repository with the dataset identifier PXD037301.
