# Supplementary material for: Stellettin B renders glioblastoma vulnerable to poly (ADP-ribose) polymerase inhibitors via suppressing homology-directed repair
Source: Signal Transduct Target Ther. 2023 Mar 22;8:119. doi: 10.1038/s41392-023-01324-8 (PMC10030553; doi:10.1038/s41392-023-01324-8)
Supplement: Supplementary file 1 — Supplementary Materials [file 41392_2023_1324_MOESM1_ESM.docx]

Supplementary Materials for

Stellettin B renders glioblastoma vulnerable to poly (ADP-ribose) polymerase inhibitors via suppressing homology-directed repair

Xin Peng^1, 2, 3, #^, Yingying Wang^1, 2, #^, Shaolu Zhang^1, 2, #^, Zhennan Tao^4^, Yuxiang Dai^4^, Francois X. Claret^3^, Moshe Elkabets^5^, Hou-Wen Lin^6, *^, Zhe-Sheng Chen^7, *^, Dexin Kong^1, 2, *^

^1^ Tianjin Key Laboratory of Technologies Enabling Development of Clinical Therapeutics and Diagnostics, School of Pharmacy, Tianjin Medical University, Tianjin 300070, China

^2^ Key Laboratory of Immune Microenvironment and Diseases (Ministry of Education), Tianjin Medical University, Tianjin 300070, China

^3^ Department of Systems Biology, The University of Texas MD Anderson Cancer Center, Houston, TX 77030, USA

^4^ Department of Neurosurgery, the Affiliated Drum Tower Hospital, School of Medicine, Nanjing University, Nanjing 210008, China

^5^ The Shraga Segal Department of Microbiology, Immunology and Genetics, Faculty of Health Sciences, Ben-Gurion University of the Negev, Beer-Sheva 84105, Israel

^6^ Research Center for Marine Drugs, State Key Laboratory of Oncogenes and Related Genes, Department of Pharmacy, Renji Hospital, School of Medicine, Shanghai Jiaotong University, Shanghai 200127, China

^7^ Department of Pharmaceutical Sciences, College of Pharmacy and Health Sciences, St. John's University, Queens, NY 11439, USA

# Xin Peng, Yingying Wang, and Shaolu Zhang contributed equally to this work.

Corresponding authors: Hou-Wen Lin franklin67@126.com; Zhe-Sheng Chen chenz@stjohns.edu; Dexin Kong kongdexin@tmu.edu.cn

**This PDF file includes:**

Materials and Methods

Supplementary Figures S1 to S8

**Materials and Methods**

**Reagents and antibodies**

We isolated STELB from *Jaspis stellifera*. TMZ was obtained from Selleck Chemicals. The antibodies for PARP, Caspase-3, Cleaved Caspase-3, phospho-Akt, PI3Kα, Ki-67, β-actin, HA-Tag, anti-rabbit IgG (H+L), F(ab')_2_ Fragment, and HRP-linked antibody were purchased from Cell Signaling Technology. The antibodies for γ-H2AX, BRCA1, BRCA2, and RAD51 were obtained from Abcam.

**Cell culture and transfection**

U251 and U87 were purchased from the Chinese Academy of Sciences (Shanghai, China). SF295 was a gift from the National Cancer Institute (NCI, USA). SHG140 are primary cells of GBM isolated from fresh tumor tissues. Cells were cultured in DMEM or RPMI 1640 containing 10% FBS (Gibco), penicillin (100 U/ml), and streptomycin (100 μg/ml). For the overexpression of PIK3CA in GBM cells, cells were transfected with HA-PIK3CA (Hanbio Biotechnology) with the Lipofectamine 3000 (Invitrogen).

**Protein extraction and digestion**

Cells were treated with DMSO (Control) or STELB (0.03 μM). After 24 hours, the total proteins were harvested. 50 μg of protein sample was denatured by adding one-fourth 8M urea. Then, 50 mM of ammonium bicarbonate was added to make the total volume reach 100 μl. Subsequently, the protein sample was exposed to 1 μl of 200 mM Dithiothreitol (DTT) for 30 min at 60°C, and then alkylated with 1 μl of 500 mM iodoacetamide. After digestion with trypsin (4 μl, 0.25 μg/μl) at 37°C overnight, the samples were desalted with a C18 SPE column (Millipore) and vacuum-dried, to be available for MS analysis.

**LC-MS/MS Analysis**

The digested peptide samples were examined with a Q Exactive Plus mass spectrometer and an EASY nano Liquid chromatography (EASY nLC 1200, Thermo Scientific). The mass spectra were searched against the UniProt database, and the raw data for each sample were searched using Maxquant (Version 2.0.3.1). Searches were carried out with trypsin cleavage specificity allowing two miscleavage events. The identification of protein was based on identification of at least one unique peptide, and the protein quantification was calculated as the median of unique peptides of the protein.

**Cell viability and synergy assay**

Cell viability was measured by CCK-8 assay. Cells were incubated with the indicated concentrations of Olaparib/Rucaparib and/or STELB for 96 hours. Then the CCK-8 (Beyotime) was added to each well. The absorbance was measured at 450 nm with an iMark microplate reader (Bio-Rad) after 2 hours. For the synergy assays, the proliferation of the cells in the different treatment groups was presented by comparison with that of control (DMSO-treated) cells, and the growth inhibition percentage was calculated. ZIP Synergy scores were calculated with the software of Synergyfinder 3.0.

**Colony formation assay**

Cells were plated at a density of 5×10^2^ cells/well, and then treated with the indicated concentrations of STELB in combination with Olaparib/Rucaparib the next day. After 72 hours, cells were further recovered in fresh medium for 10 days and then stained with 0.25% crystal violet (Sigma-Aldrich).

**Apoptosis assay**

Cells were exposed to Olaparib/Rucaparib and/or STELB. After 48 hours of treatment, the cells were resuspended in binding buffer containing Annexin V/PI (BD Biosciences) in the dark for 15 min. The apoptosis was examined with FACS Verse Flow Cytometer (BD Biosciences) and quantified with FlowJo Software (Tristar).

**Alkaline comet assay**

After treated with Olaparib/Rucaparib and/or STELB, cells were embedded and solidified in low melting point agarose. Slides were immersed in lysis solution overnight at 4 °C and then transferred to the electrophoretic box. After electrophoresis, the slides were stained with SYBR Gold (Invitrogen). Cells were randomly analyzed using CASP software (CaspLab). The % DNA in tails is expressed as the extent of DNA damage.

**Immunofluorescence staining**

Cells were incubated on coverslips in 24-well plates in the presence of Olaparib/Rucaparib and/or STELB. Then, cells were fixed and blocked, and stained with the γ-H2AX or RAD51 primary antibody overnight at 4 °C. The next day, coverslips were washed and incubated with the respective secondary antibody for 1 hour. Finally, nuclei were stained using DAPI. An Olympus BX51 fluorescence microscope was used to capture images.

**siRNA knockdown**

Cells were cultured in 6-well plates and transfected with siRNA targeting PIK3CA (5'-CUGAGAAAAUGAAAGCUCACUCUTT-3') (Sigma-Aldrich) or a non-targeting siRNA (siNT) using the lipofectamine 3000 transfection reagent (Invitrogen), following the manufacturer’s protocol.

**qRT-PCR**

Total RNA was isolated from cell cultures using an RNeasy Mini kit (Qiagen). Two micrograms of the RNA samples was reversetranscribed using a cDNA Synthesis Kit (GenStar). RT-PCR was done with aliquots of cDNA samples mixed with SYBR Green Master Mix (Applied Biosystems). Reactions were performed in triplicate. The fold differences in transcripts were calculated using the ΔΔCt method with 18S rRNA as a control. The following primers were used: BRCA1 forward, 5′- GAACGGGCTTGGAAGAAAAT-3′; BRCA1 reverse, 5′- GTTTCACTCTCACACCCAGA-3′; BRCA2 forward, 5′- CAGGTAGACAGCAGCAAGCA-3′; BRCA2 reverse, 5′- AAGCCCCTAAACCCCACTTC-3′; RAD51 forward, 5′- CAGATGCAGCTTGAAGCAAA-3′; RAD51 reverse, 5′- TTCTTCACATCGTTGGCATT-3′; 18S rRNA forward, 5′- CAGCCACCCGAGATTGAGCA-3′; 18S rRNA reverse, and 5′-TAGTAGCGACGGGCGGTGTG-3′.

**Western blots**

Cells were seeded in 6-well plates at a density of 40% confluence. After 24 hours, the cells in each well were exposed to Olaparib/Rucaparib and/or STELB. Then, cells were lysed in RIPA buffer. Total protein (20-50 µg) for each sample was loaded onto an SDS-PAGE gel and transferred to polyvinylidene fluoride membranes (Bio-Rad). After blocking in 5% nonfat dry milk, the membranes were exposed to primary antibodies overnight at 4°C, and then to the respective HRP-conjugated secondary antibodies for 1 hour. The signals were visualized with an ECL detection kit (Bio-Rad), and β-actin was used as the loading control.

**DR-GFP reporter assay**

*In vitro* cell transfections were performed in 6-well plates with the HDR repair reporter substrate direct repeat GFP (DR-GFP) plasmid and the pCBASceI plasmid/empty vectors, and the lipofectamine 3000transfection reagent (Invitrogen). GFP-expressing plasmid (pEGFP-C1) served as a transfection efficiency control. After 6 hours, the cells were incubated with Rucaparib and/or STELB, then collected and resuspended in PBS after 48 hours. The GFP intensity was detected with a FACS Verse flow cytometer (BD Biosciences).

**EJ5-GFP reporter assay**

*In vitro* cell transfections were performed in 6-well plates with EJ5-GFP plasmid and the pCBASceI plasmid/empty vector, and the lipofectamine 3000 transfection reagent (Invitrogen). GFP-expressing plasmid (pEGFP-C1) served as a transfection efficiency control. Other methods for sample preparation and analysis are the same as described in DR-GFP reporter assay.

**Heterotopic nude mouse xenograft model**

All animal experiments were carried out in accordance with the National Institutes of Health Guide for the Care and Use of Laboratory Animals. U87 cells were subcutaneously injected into the right lateral flank of male BALB/c nude mice (Vital River Laboratory Animal Technology Company, Beijing, China). Then, the animals were randomly divided into the following treatment groups: Experiment 1: vehicle, STELB (1 mg/kg), STELB (2 mg/kg), STELB (4 mg/kg), TMZ (6 mg/kg) (positive control); Experiment 2: vehicle, STELB (2 mg/kg), Rucaparib (4 mg/kg), STELB and Rucaparib (with the same doses as every single agent). Tumor size was measured every three days until the endpoint. The formula (length × width^2^)/2 was used to calculate the tumor volume. The mice were euthanized at the end of the experiments, and the tumors were excised and fixed for histological analysis.

**H&E, TUNEL, and immunohistochemical (IHC) staining**

H&E staining was conducted to detect pathological changes. Apoptotic cells in tumors were detected using a TUNEL Apoptosis Detection Kit (Beyotime). Paraffin-embedded tumor tissues were sectioned, hydrated, and subjected to heat-induced antigen retrieval. Then, the sections were exposed to primary antibodies overnight at 4°C and subsequently to Envision anti-Rabbit (DAKO), followed by visualization with DAB reagent (GeneTech). Then, the sections were counterstained using hematoxylin and mounted. Images were taken using the high-resolution digital slide scanner (Pannoramic 250, 3DHistech).

**Orthotopic zebrafish xenograft model**

Wild-type AB zebrafish (*Danio rerio*) were kept at 28°C in a controlled multi-tank recirculating system. Two days after fertilization, embryos were anesthetized using 1.2 mM tricaine and put onto a modified agarose gel mold for cancer cell microinjection. A total of 50-100 U87-RFP cells (in 5 nl of serum-free culture medium) were injected into the brains of zebrafish larvae using a pneumatic pico-pump injector. The injected embryos were selected and separately removed to a 48-well plate containing different drugs in 2 ml of E3 media and incubated at 32°C for 4 days. Xenografts were observed under an inverted microscope (IX71, Olympus) every two days, and the fluorescence intensity was determined with the software of ImageJ.

**Orthotopic nude mouse xenograft model**

U87-Luc cells were injected into the right striatum of 4- or 5- week-old male BALB/c nude mice. Using an IVIS luminescent imaging system (IVIS Spectrum, PerkinElmer), tumors were measured after 10 days of injection and randomly divided into the following 4 groups: vehicle, STELB (2 mg/kg), Rucaparib (4 mg/kg), combination of STELB and Rucaparib. Tumors were measured by luminescence imaging at indicated durations. Images were analyzed with the Living Image software (PerkinElmer).

**Statistical analysis**

Experiments were performed for at least 3 times as described in the figures and corresponding figure legends. Data were expressed as mean ± SEM. Student’s t-test was used when two groups were compared. ANOVA with Bonferroni’s correction was used to compare multiple groups. *p < 0.05, **p < 0.01, ***p < 0.001, ****p < 0.0001.


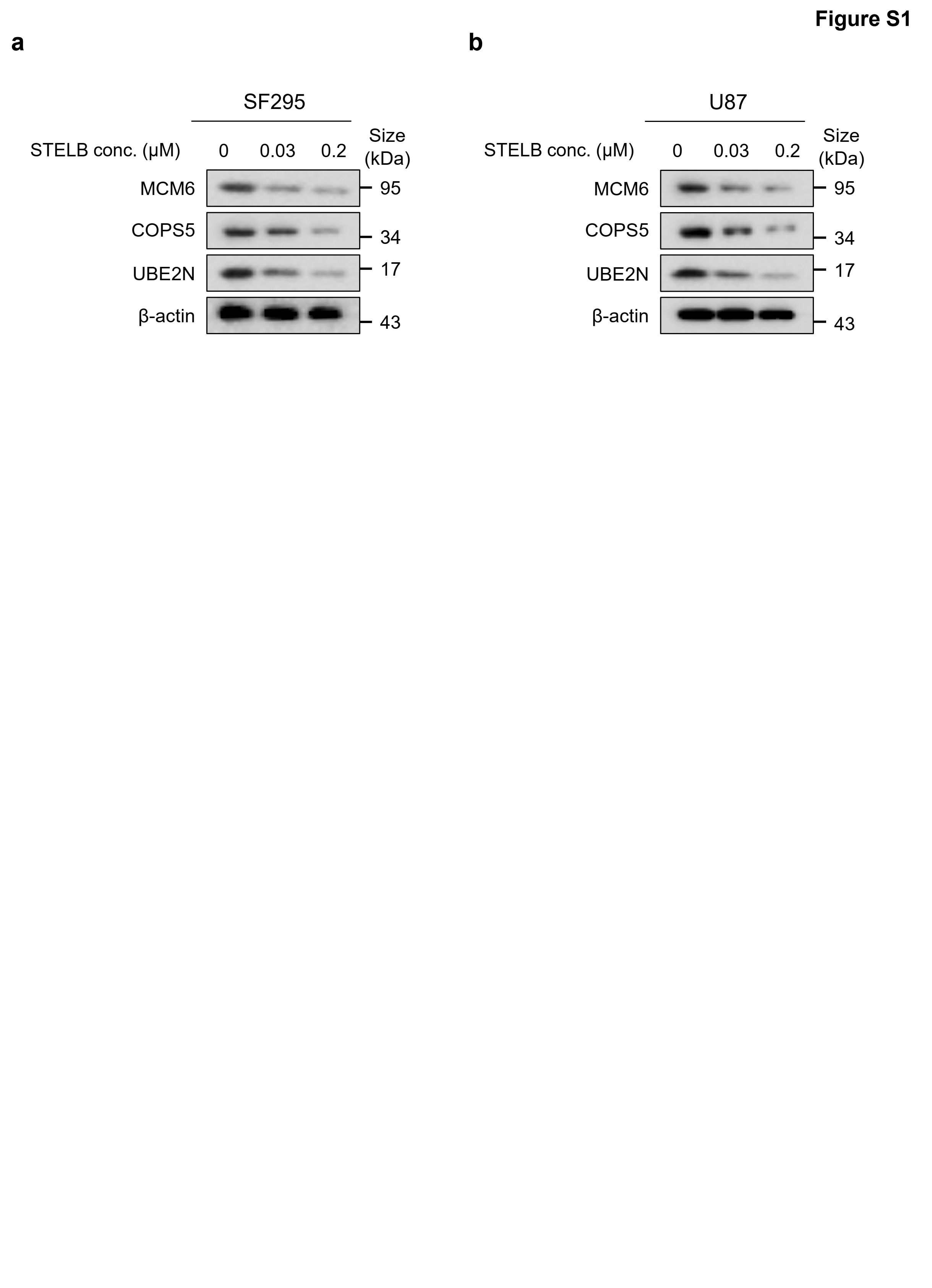


**Supplementary Figure 1.** **Validation of mass spectrometry data.** Western blot analysis of the representative DNA repair-related proteins in SF295 (**a**) and U87 (**b**) cells in the presence of STELB, of which similar changes were found in mass spectrometry data.

**
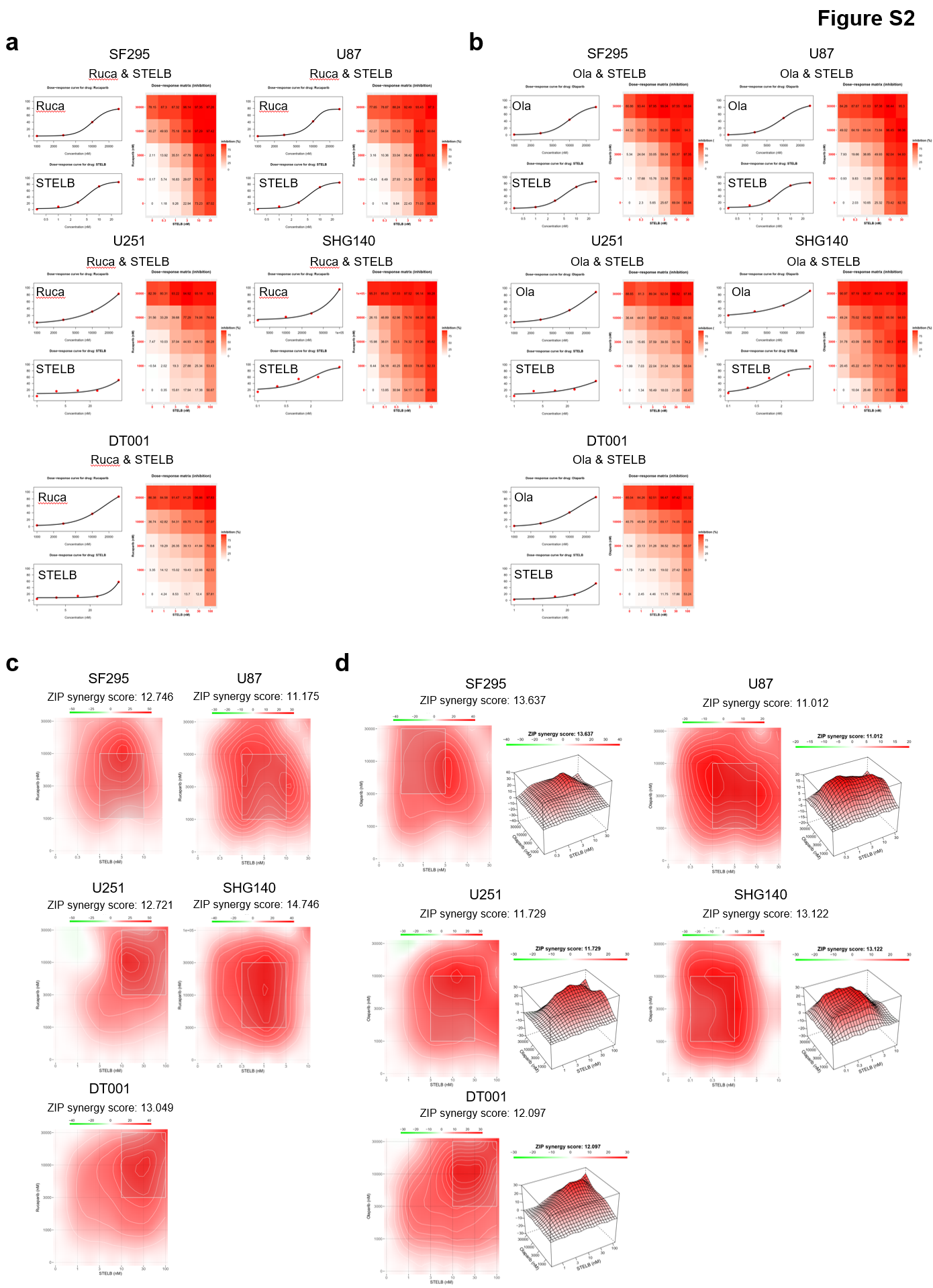
Supplementary Figure 2.** **ZIP model synergy test in GBM cells.** (**a, b**) Dose-response curve and Dose-response matrix of Olaparib/Rucaparib and STELB in established cell lines of GBM (SF295, U87, and U251), and primary patient-derived cell lines of GBM (SHG140, DT001). (**c, d**) the ZIP synergy score of combination of STELB and Rucaparib/Olaparib.


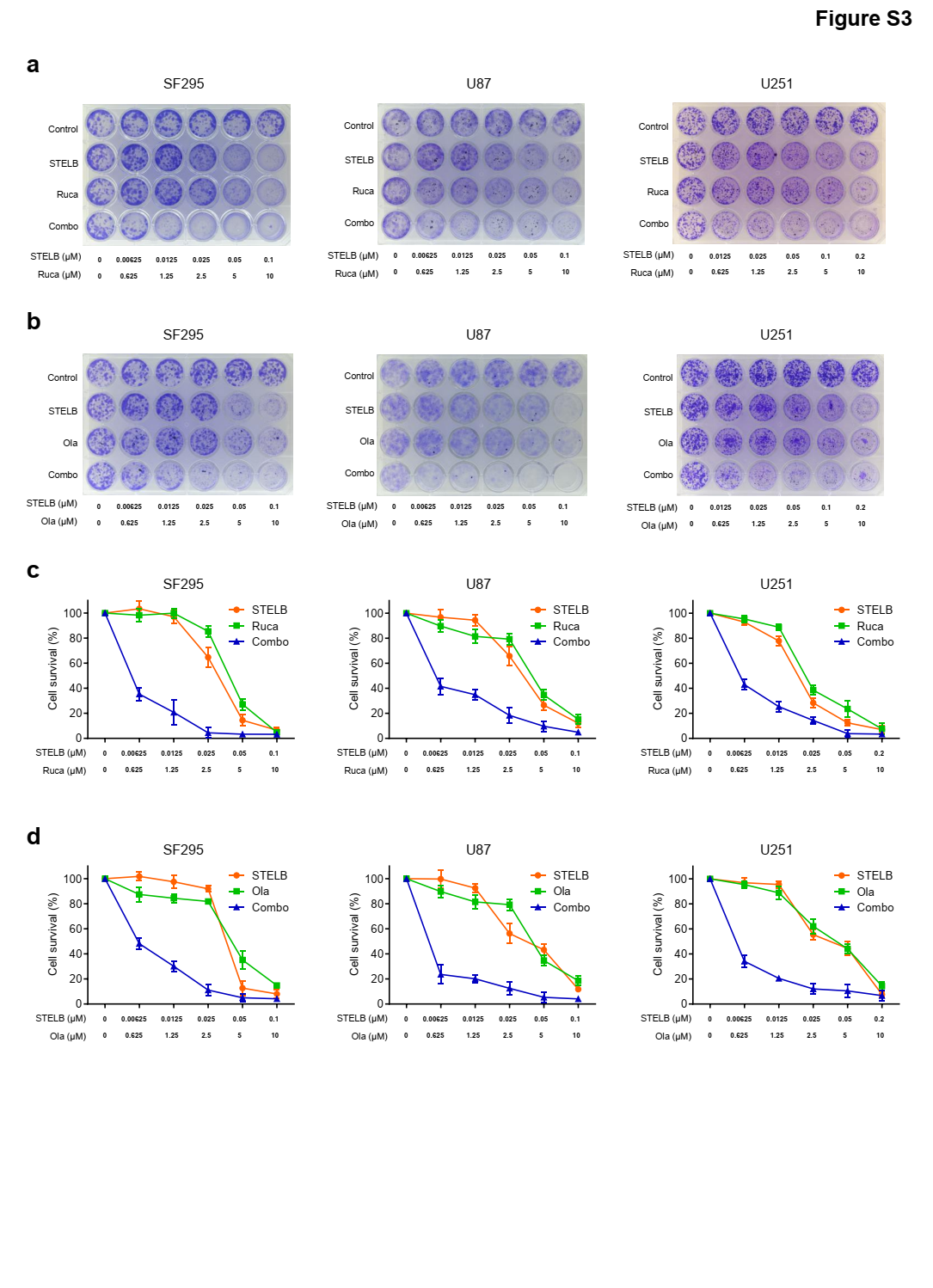


**Supplementary Figure 3. STELB synergizes with PARPis to inhibit colony forming abilities of GBM cells.** (**a, b**) Clonogenic assays of GBM cells treated with Olaparib/Rucaparib and/or STELB for 72 hours, and allowed to recover in the fresh media for 10 days. Then the cells were subjected to crystal violet (0.25%) staining. (**c, d**) Quantification of (**a, b**), after incubation with 1% SDS. The absorbance at 570 nm was detected.


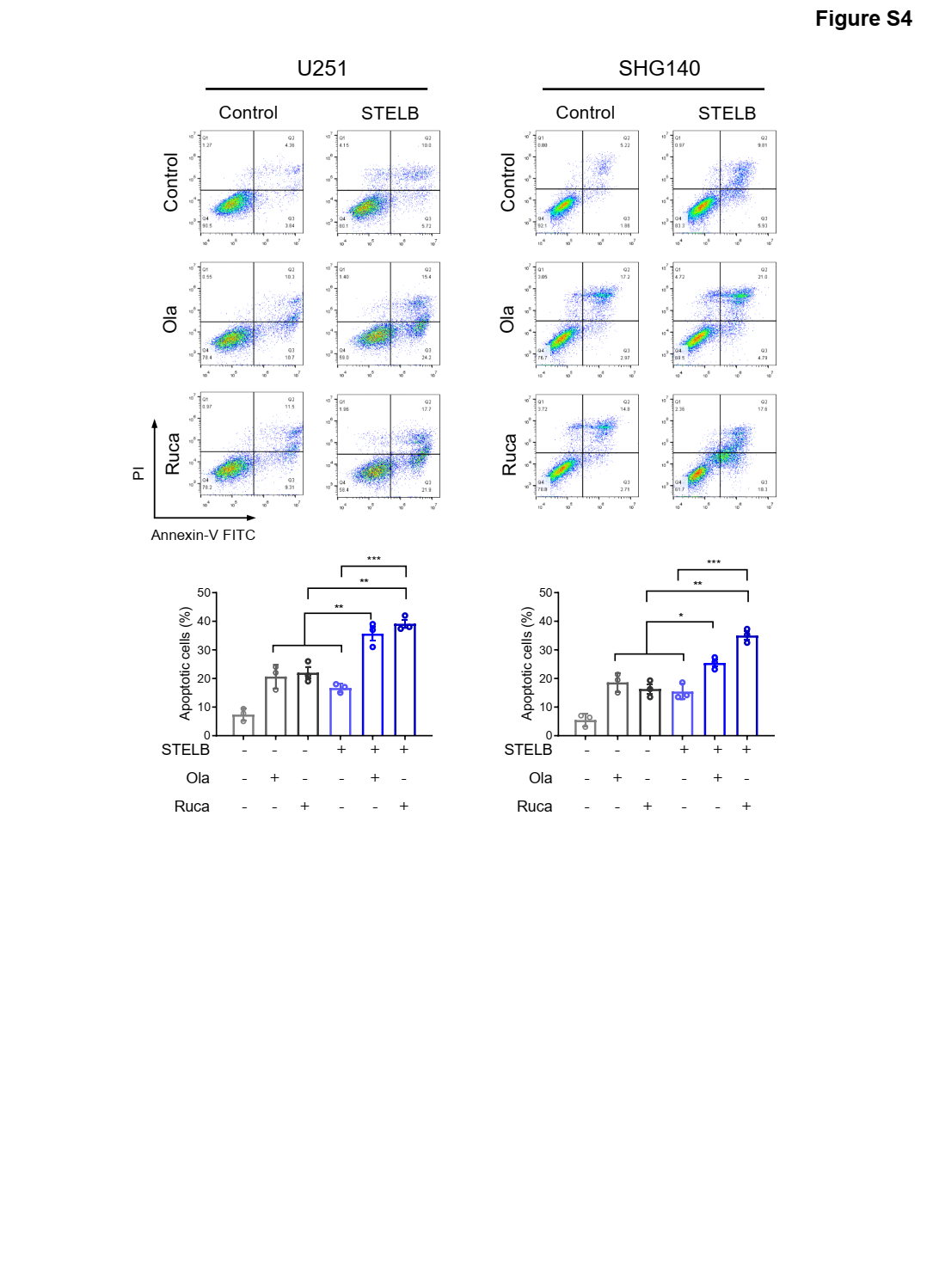


**Supplementary Figure 4. STELB synergizes with PARPis to induce apoptosis in U251 and SHG140 cells.** U251 and SHG140 cells were incubated with STELB in combination with Olaparib/Rucaparib for 48 hours. Then the cells were subjected to apoptosis assays. FACS quantification of the total apoptotic cell population, including Annexin V^+^/PI^−^ early apoptotic cells and Annexin V^+^/PI^+^ late apoptotic cells. Quantitative data are represented as mean ± SEM from at least 3 independent experiments; *P < 0.05, **P < 0.01, ***P < 0.001.


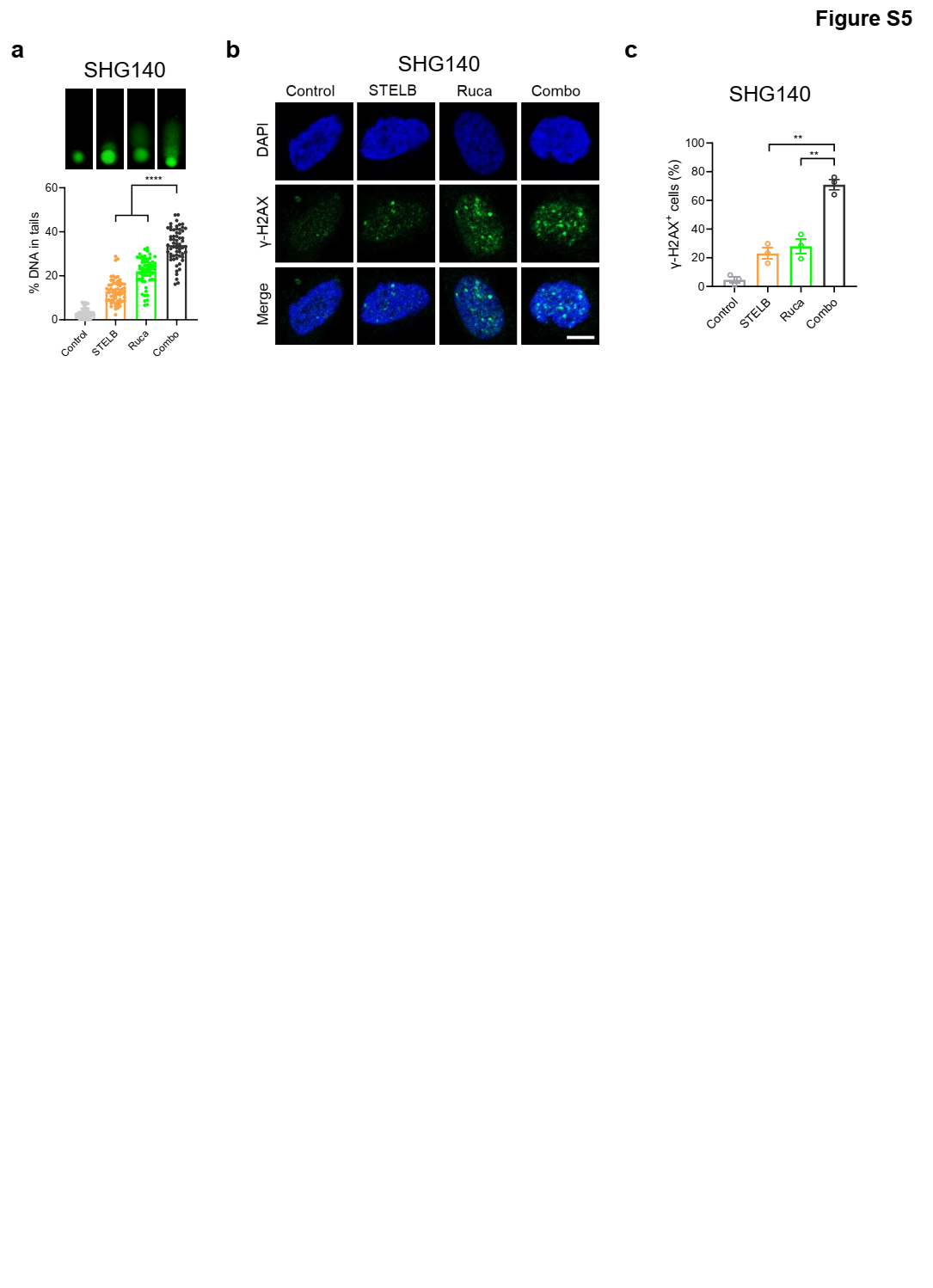


**Supplementary Figure 5. STELB synergizes with PARPis to induce DSBs in SHG140 cells.** (**a**) SHG140 cells were incubated with Rucaparib and/or STELB for 48 hours. Then the cells were subjected to alkaline comet assays. The % DNA in tails was calculated to reflect the extent of DNA damage. (**b**) SHG140 cells were incubated with Rucaparib and/or STELB for 48 hours. Then the cells were subjected to γ-H2AX foci assays. Scale bar, 20 μm. (**c**) The percentage of γ-H2AX foci positive cells (> 5 foci/cell) is shown. Quantitative data are indicated as mean ± SEM of at least 3 independent experiments; **P < 0.01, ****P < 0.0001.


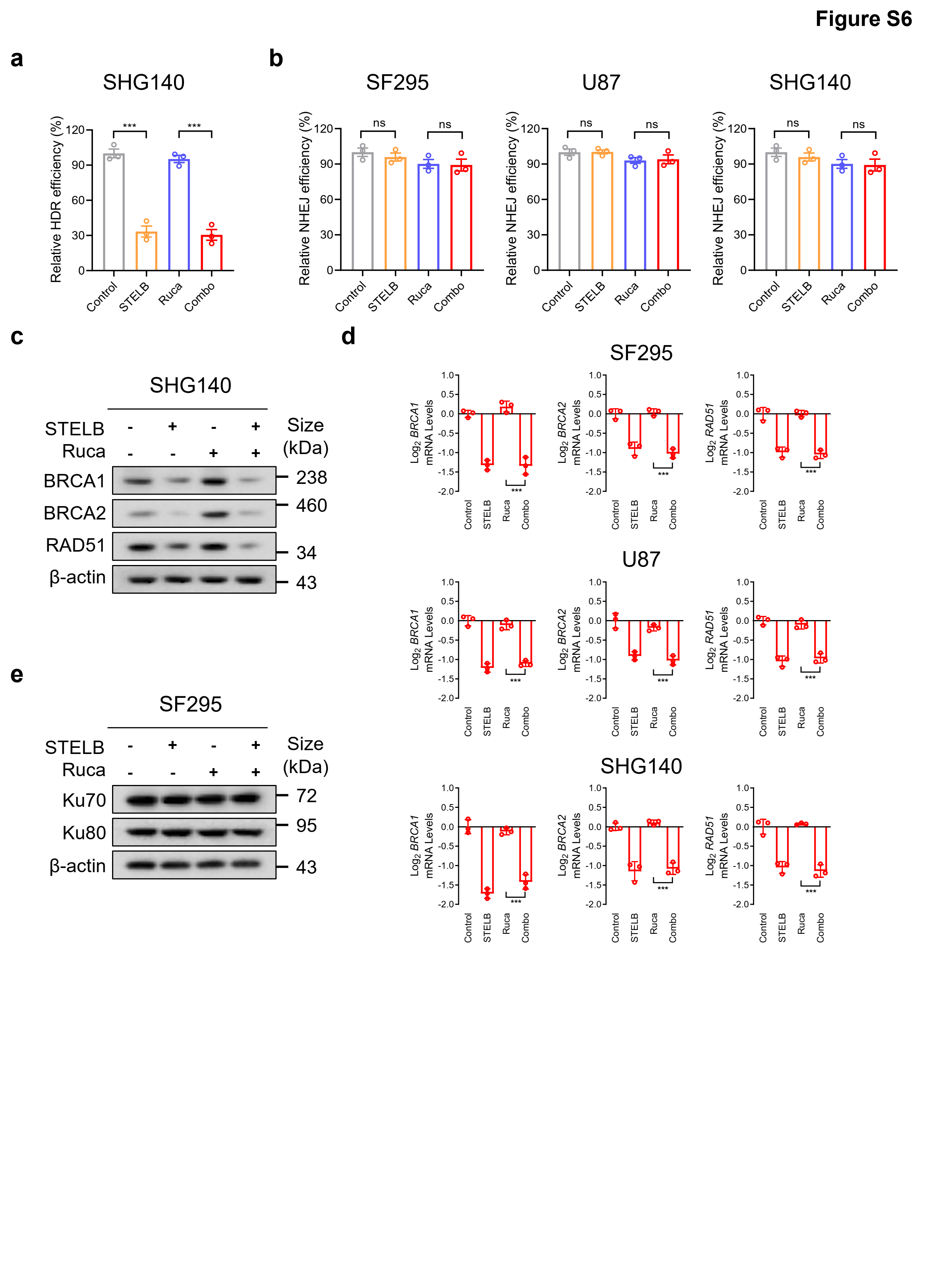


**Supplementary Figure 6. STELB suppresses HDR in GBM cells.** (**a**) DR-GFP reporter assay was used to evaluate HDR repair efficiency in SHG140 cells. (**b**) EJ5-GFP reporter assays to evaluate NHEJ repair efficiency in SF295, U87, and SHG140 cells. (**c**) Western blot analysis of HDR-related proteins in SHG140 cells incubated with Rucaparib and/or STELB for 48 hours. (**d**) qRT-PCR analysis of HDR-related genes in GBM cells incubated with Rucaparib and/or STELB for 48 hours. (**e**) Western blot detection of Ku70 and Ku80 in SF295 cells incubated with Rucaparib and/or STELB for 48 hours. Quantitative data are represented as mean ± SEM of at least 3 independent experiments; ns, not significant, ***P < 0.001.


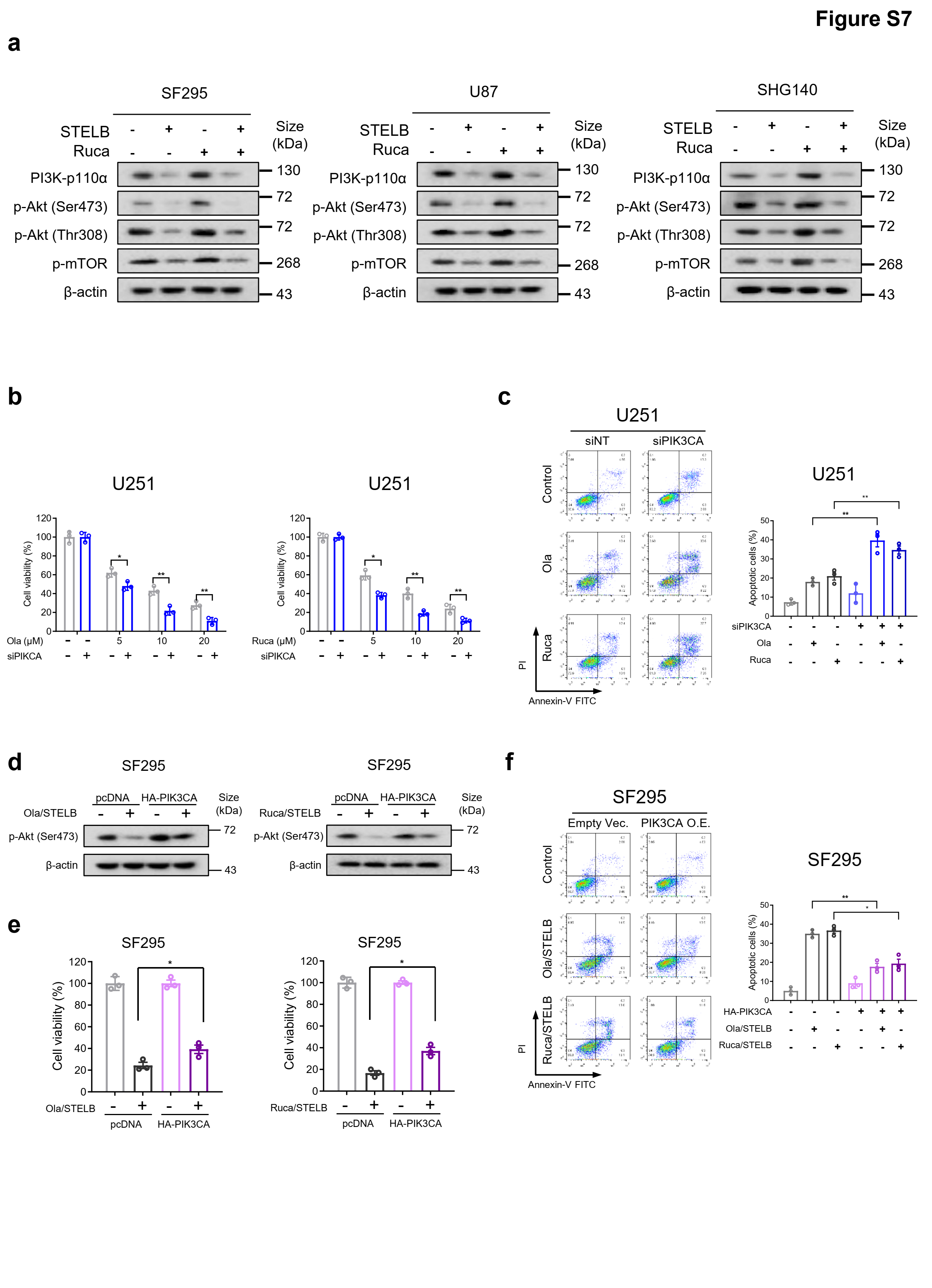


**Supplementary Figure 7. STELB sensitizes PARPis through the inhibition of PI3K in GBM cells.** (**a**) Western blot detection of PI3K-p110α, p-Akt (Ser473), p-Akt (Thr308), and p-mTOR in SF295, U87, and SHG140 cells incubated with Rucaparib and/or STELB for 48 hours. (**b, c**) Cell viability assays and apoptosis assays of U251 cells incubated with Olaparib or Rucaparib with or without siPIK3CA. (**d**) Western blot detection of p-Akt in SF295 cells incubated with STELB and/or Olaparib/Rucaparib in the presence or absence of HA-PIK3CA. (**e, f**) Cell viability assays and apoptosis assays SF295 cells treated with STELB and Olaparib/Rucaparib with or without HA-PIK3CA. Quantitative data are indicated as mean ± SEM of at least 3 independent experiments; *P < 0.05, **P < 0.01.


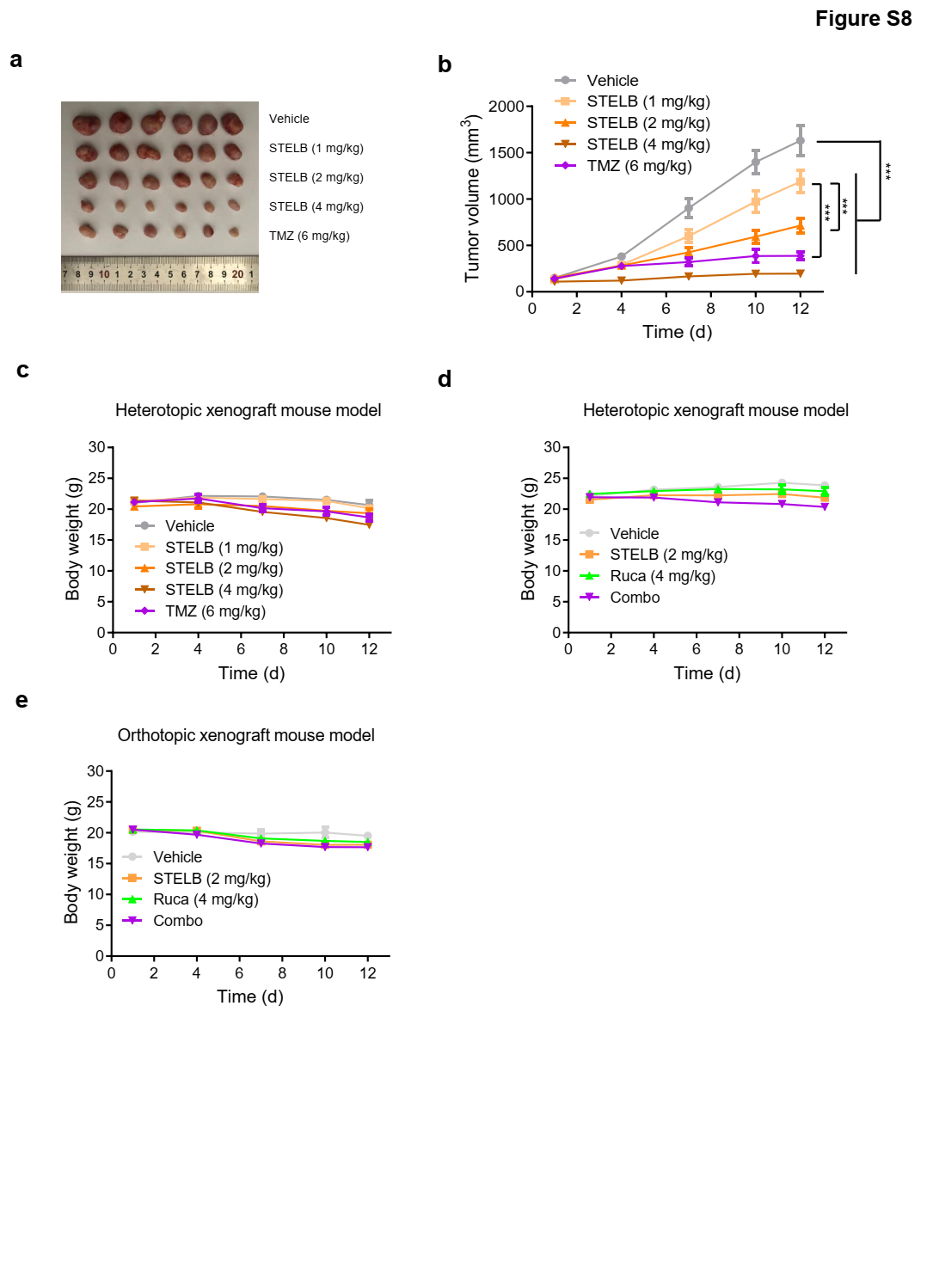


**Supplementary Figure 8. STELB sensitizes PARPis through the inhibition of PI3K in GBM cells.** (**a**) Nude mice with subcutaneous U87 tumors were administrated with placebo, increasing doses of STELB (1, 2, 4 mg/kg), or the positive control temozolomide (TMZ) (6 mg/kg) for 12 days (n = 5). Excised tumors are shown. Tumor volumes (**b**) and body weight (**c**) were measured every 3 days. (**d**) Body weight was measured in the group of vehicle, Rucaparib (4 mg/kg), STELB (2 mg/kg), or the Rucaparib and STELB combination in heterotopic xenograft tumor model mice for 12 days (n = 5). (**e**) Body weight was measured in the group of vehicle, Rucaparib (4 mg/kg), STELB (2 mg/kg), or the Rucaparib and STELB combination in orthotopic xenograft tumor model mice for 12 days (n = 5). Quantitative data are indicated as mean ± SEM of at least 3 independent experiments; ***P < 0.001.
